# Prognostic Impact of Renal Dysfunction Does Not Differ According to the Clinical Profiles of Patients: Insight from the Acute Decompensated Heart Failure Syndromes (ATTEND) Registry

**DOI:** 10.1371/journal.pone.0105596

**Published:** 2014-09-08

**Authors:** Taku Inohara, Shun Kohsaka, Naoki Sato, Katsuya Kajimoto, Takehiko Keida, Masayuki Mizuno, Teruo Takano, on behalf of the ATTEND Investigators

**Affiliations:** 1 Department of Cardiology, Keio University School of Medicine, Tokyo, Japan; 2 Internal Medicine, Cardiology and Intensive Care Unit, Nippon Medical School Musashi-Kosugi Hospital, Kanagawa, Japan; 3 Department of Cardiology, Sensoji Hospital, Tokyo, Japan; 4 Department of Cardiology, Edogawa Hospital, Tokyo, Japan; 5 Department of Cardiology, Tokyo Women's Medical University, Tokyo, Japan; 6 Department of Internal Medicine, Nippon Medical University, Tokyo, Japan; I2MC INSERM UMR U1048, France

## Abstract

**Background:**

Renal dysfunction associated with acute decompensated heart failure (ADHF) is associated with impaired outcomes. Its mechanism is attributed to renal arterial hypoperfusion or venous congestion, but its prognostic impact based on each of these clinical profiles requires elucidation.

**Methods and Results:**

ADHF syndromes registry subjects were evaluated (N = 4,321). Logistic regression modeling calculated adjusted odds ratios (OR) for in-hospital mortality for patients with and without renal dysfunction. Renal dysfunction risk was calculated for subgroups with hypoperfusion-dominant (eg. cold extremities, a low mean blood pressure or a low proportional pulse pressure) or congestion-dominant clinical profiles (eg. peripheral edema, jugular venous distension, or elevated brain natriuretic peptide) to evaluate renal dysfunction's prognostic impact in the context of the two underlying mechanisms. On admission, 2,150 (49.8%) patients aged 73.3±13.6 years had renal dysfunction. Compared with patients without renal dysfunction, those with renal dysfunction were older and had dominant ischemic etiology jugular venous distension, more frequent cold extremities, and higher brain natriuretic peptide levels. Renal dysfunction was associated with in-hospital mortality (OR 2.36; 95% confidence interval 1.75–3.18, p<0.001), and the prognostic impact of renal dysfunction was similar in subgroup of patients with hypoperfusion- or congestion-dominant clinical profiles (p-value for the interaction ranged from 0.104–0.924, and was always >0.05).

**Conclusions:**

Baseline renal dysfunction was significantly associated with in-hospital mortality in ADHF patients. The prognostic impact of renal dysfunction was the same, regardless of its underlying etiologic mechanism.

## Introduction

Despite advances in pharmacological and mechanical therapies, acute decompensated heart failure (ADHF) remains one of the most frequently encountered and life-threatening cardiovascular conditions [1]. The EuroHeart Failure Survey, which included 11,327 patients with ADHF, showed that post-discharge mortality rates reached 8.1% and 20.5% within 3 months and 1 year, respectively [2].

Baseline renal dysfunction is one of the most important predictors of short- and long-term cardiovascular outcomes in patients with ADHF [3]–[5]. Although several mechanisms coexist in the deterioration of renal function among ADHF patients [6], [7], two hemodynamic mechanisms, renal arterial hypoperfusion and renal venous congestion, broadly describe the processes underlying renal dysfunction. Traditionally, renal dysfunction associated with ADHF has been attributed to hypoperfusion of the kidney caused by the progressive impairment of cardiac output [8]. However, recent studies have demonstrated that hypotension is rarely observed in patients with renal dysfunction [9], and that the elevation of central venous pressure (CVP) is more closely associated with worsening renal function than the cardiac index [10]. This suggests that in patients with ADHF admitted to hospital, renal dysfunction is more dependent on venous congestion than on the impairment of cardiac output.

The contributions of renal hypoperfusion and congestion to renal dysfunction have not been thoroughly investigated. Hemodynamic profiles can be assessed by measuring blood pressure, performing physical examinations, and by measuring laboratory markers [11], [12], and these parameters are used to assess the mechanisms underlying renal dysfunction. This study aimed to clarify differences in the prognostic impact of renal dysfunction on in-hospital mortality in patients admitted with ADHF, based on the underlying hemodynamic mechanisms.

## Methods

### Data sources

The study was conducted in accordance with the Declaration of Helsinki and the Japanese ethical guidelines for clinical studies. The study protocol was registered to the University Hospital Medical Information Network (UMIN 000000736), and approved by the ethics committee at each site.

The Acute Decompensated Heart Failure Syndromes (ATTEND) registry is a nationwide, multicenter, prospective cohort study that focuses on ADHF in Japan. The details of this cohort study have been reported previously [13]. In brief, patients hospitalized for ADHF who met the modified Framingham criteria, were eligible for the study. The ATTEND registry enrolled patients from April 2007 to December 2011 in 52 hospitals throughout Japan. Approximately 200 variables were collected on admission for each patient, and clinical variables included the patient's history, physical examination results, echocardiographic data, and laboratory data. Patients aged <20 years and those not considered suitable for the study by attending physicians were excluded. The present study also ruled out acute coronary syndrome. In-hospital mortality was defined as (1) death from any cause, (2) death from cardiac causes, including sudden cardiac death and heart failure death, and (3) death from cerebral or vascular causes. Death was considered cardiac-related (defined as heart failure death, sudden death, or other cardiac death), unless a specific non-cardiac cause was identified by the primary physicians. The end-point classification committee, comprising two experienced cardiologists who were not study investigators, reviewed the data and, if any problems were encountered, they asked the primary physician to confirm the cause of death. Finally, the committee categorized each event for use in the present analysis. All data are managed at an independent biostatistics and data center (STATZ Institute, Inc., Tokyo, Japan). In this study, the data was collected from multiple institutions in Japan, and the IRB approval was obtained individually from each sites. Therefore, the full set of data cannot be made available to public. The reader may contact the corresponding author to request the data.

### Study population

After excluding patients who were on hemodialysis or who had stage 5 chronic kidney disease (defined as an estimated glomerular filtration rate [eGFR] <15 mL/min/1.73 m^2^) and were supported by intracardiac balloon pumping or percutaneous cardiopulmonary support, the remaining 4,321 subjects were analyzed in this study.

### Evaluation of renal function

**Figure 1:**
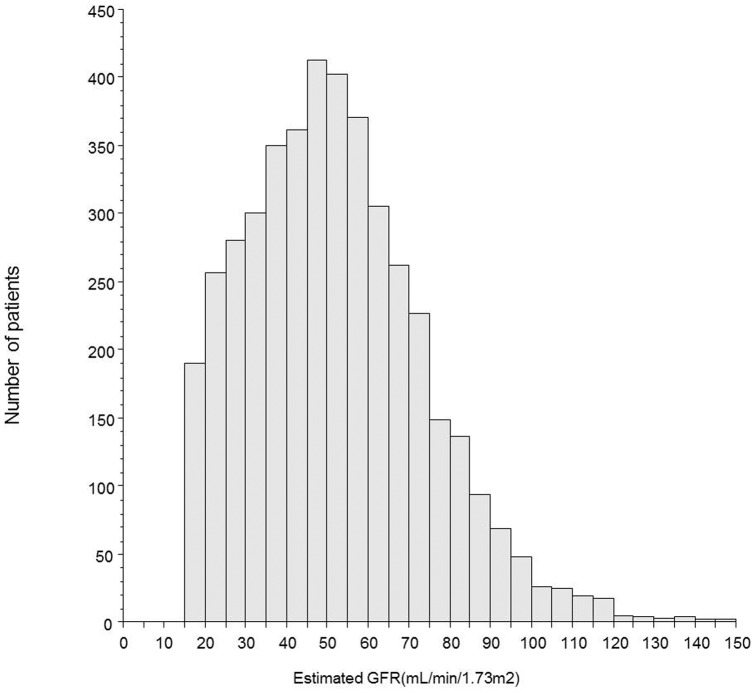
Distribution of estimated glomerular filtration rates levels on admission to hospital. GFR, glomerular filtration rate

**Figure 2:**
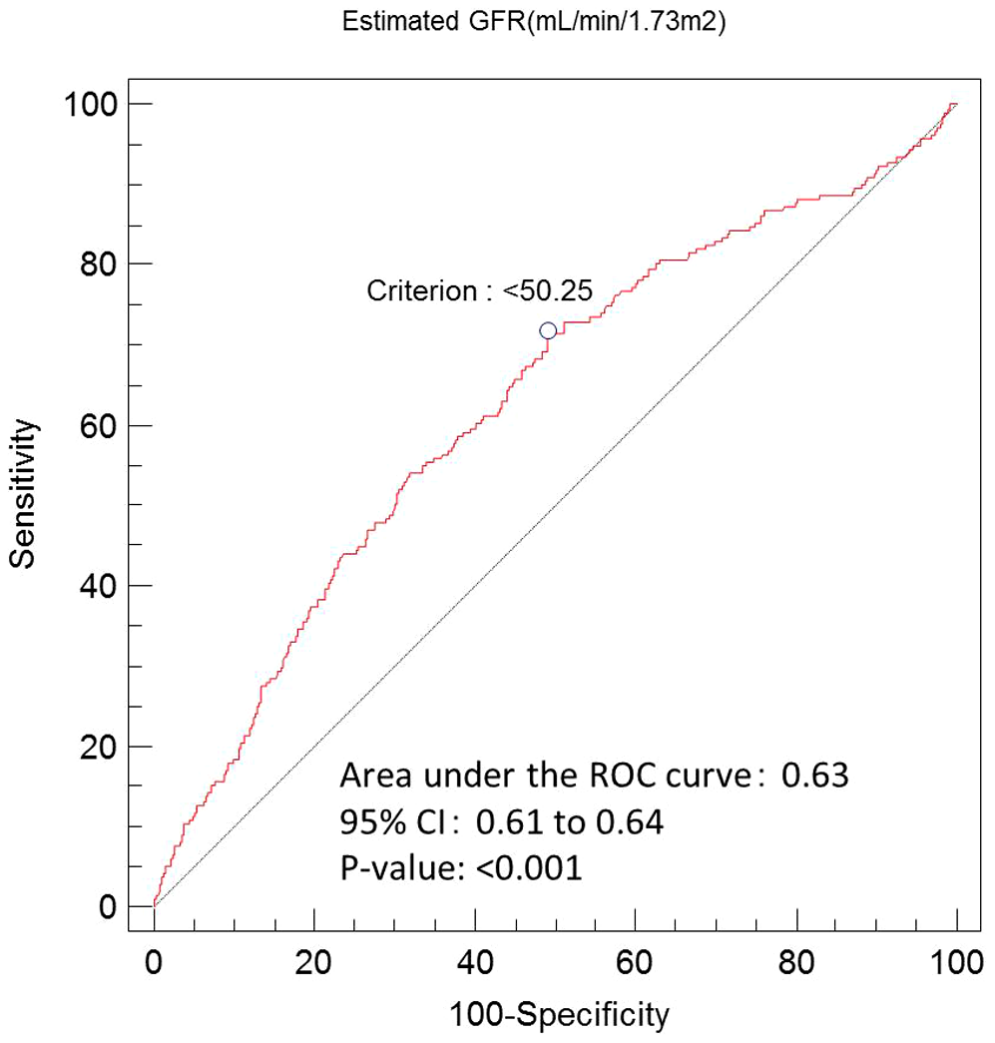
Evaluation of the receiver operating characteristic curve for renal dysfunction. The area under the curve was 0.63 (95% confidence interval = 0.61–0.64, p<0.001), and the cut-off value for the greatest sensitivity and specificity was 50.25 mL/min/1.73 m^2^. GFR, glomerular filtration rate; CI, confidence interval; ROC, receiver operating characteristic.

The National Kidney Foundation advocates estimating the GFR by using the Modification of Diet in Renal Disease (MDRD) formula to detect the early stages of renal dysfunction [14]. On the basis of this recommendation, renal function in this study was evaluated by estimating the GFR, which was calculated using the abbreviated MDRD study equation:
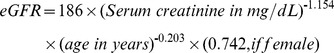
The distribution of eGFRs is shown in Figure 1. An evaluation of the receiver operating characteristic curve determined that the optimal cut-off value for renal dysfunction was estimated as a GFR ≤50 mL/min/1.73 m^2^ (Figure 2), and the area under the curve was 0.63 (95% confidence interval [CI] = 0.61–0.64, p<0.001).

### Assessing renal dysfunction mechanisms

Renal dysfunction as it relates to hypoperfusion, which is usually caused by a low-output status, was defined as the presence of cold extremities, a low left ventricular ejection fraction (LVEF) of ≤40%, a low mean blood pressure (mBP) of ≤100 mmHg [15], or a low proportional pulse pressure (PPP) of ≤40% [16]. In contrast, renal dysfunction as it relates to congestion was defined as the presence of peripheral edema or jugular venous distension (JVD), or elevated brain natriuretic peptide (BNP) levels of >677 pg/mL [17]. The cutoff values of mBP, PPP, and BNP were determined according to the respective median values.

### Statistical analysis

All data are expressed as means ± standard deviations or medians with the interquartile ranges. The receiver operating characteristic curve for renal dysfunction was used to evaluate the optimal cut-off value. Differences in each variable between patients with and without renal dysfunction were evaluated using the chi-square test or Fisher's exact test for categorical variables, and using Student's unpaired *t*-test or Mann-Whitney U test for continuous variables. A logistic regression model was used to evaluate the influence of renal dysfunction on in-hospital mortality. In the logistic regression models, the covariates were age, gender, etiology (ischemic or non-ischemic), systolic blood pressure, and heart rate. The covariates incorporated into these models were clinically associated with in-hospital mortality in patients with ADHF.

Data analyses were performed using SAS, software version 9.1 (SAS Institute Inc., Cary, North Carolina). All p-values were two-sided, and significance was defined as p<0.05. All analyses were performed at an independent biostatistics and data center (STATZ Institute, Inc., Tokyo, Japan).

## Results

Of the 4,321 patients hospitalized with ADHF, renal dysfunction was present in 2,150 (49.8%) patients and was determined on the basis of a GFR cut-off value of ≤50 mL/min/1.73 m^2^. Table 1 presents a comparison of the demographic and baseline characteristics of patients with and without renal dysfunction. In comparison with those patients without renal dysfunction, patients with renal dysfunction were older, they were more likely to have an ischemic etiology and to have histories of hospitalization for heart failure, and they were more likely to have risk factors for cardiovascular disease, which included hypertension, dyslipidemia, and diabetes mellitus. On admission to hospital, physical findings, including JVD and cold extremities, were more frequently observed in patients with renal dysfunction than in patients without renal dysfunction. Patients with renal dysfunction had significantly lower blood pressures and heart rates, and significantly higher plasma BNP levels, compared with those without renal dysfunction.

**Table 1:** Baseline characteristics of patients with and without renal dysfunction.

	Total	eGFR >50 mL/min/1.73 m^2^	eGFR ≤50 mL/min/1.73 m^2^	
	(N = 4,321)	(n = 2,171)	(n = 2,150)	p-value
Mean age (years)	73.3±13.6	70.2±14.4	76.5±11.9	<0.001
Men, n (%)	2,501 (57.9)	1,300 (59.9)	1,201 (55.9)	0.007
Ischemic cause of HF, n (%)	1,283 (29.7)	564 (26.0)	719 (33.4)	<0.001
Medical history				
Prior hospitalization for HF, n (%)	1,521 (35.2)	576 (26.5)	945 (44.0)	<0.001
Hypertension, n (%)	2,980 (69.0)	1,417 (65.3)	1,563 (72.7)	<0.001
Dyslipidemia, n (%)	1,558 (36.1)	736 (33.9)	822 (38.2)	0.003
Diabetes mellitus, n (%)	1,391 (32.2)	667 (30.7)	724 (33.7)	0.036
Smoking, n (%)	1,840 (42.6)	990 (45.6)	850 (39.5)	<0.001
Atrial flutter or fibrillation, n (%)	1,745 (40.4)	849 (39.1)	896 (41.7)	0.096
Chronic respiratory disease, n (%)	538 (12.5)	263(12.1)	275 (12.8)	0.501
Stroke/transient ischemic attack, n (%)	611 (14.1)	261 (12.0)	350 (16.3)	<0.001
Pacemaker/ICD, n (%)	380 (8.8)	142 (6.5)	238 (11.1)	<0.001
Cardiac resynchronization therapy, n (%)	86 (2.0)	24 (1.1)	62 (2.9)	<0.001
Clinical profile on admission				
Paroxysmal nocturnal dyspnea, n (%)	2,288 (53.0)	1,161 (53.5)	1,127 (52.4)	0.609
Orthopnea, n (%)	2,717 (62.9)	1,368 (63.0)	1,349 (62.7)	0.795
Rales, n (%)	3,075 (71.2)	1,548 (71.3)	1,527 (71.0)	0.938
Third heart sound, n (%)	1,518 (35.1)	745 (34.3)	773 (36.0)	0.35
Jugular venous distension, n (%)	2,246 (52.0)	1,088 (50.1)	1,158 (53.9)	0.005
Peripheral edema, n (%)	2,887 (66.8)	1,423 (65.5)	1,464 (68.1)	0.075
Cold extremities, n (%)	917 (21.2)	409 (18.8)	508 (23.6)	<0.001
EF≤40%, n (%)	2,301 (53.3)	1,181 (54.4)	1,120 (52.1)	0.158
NYHA functional class				
I, n (%)	74 (1.7)	40 (1.8)	34 (1.6%	0.458
II, n (%)	706 (16.3)	372 (17.1)	334 (15.5)	
III, n (%)	1,657 (38.3)	825 (38.0)	832 (38.7)	
IV, n (%)	1,834 (42.4)	909 (41.9)	925 (43.0)	
Mean heart rate (beats/min)	99.0±29.3	102.9±29.4	95.0±28.7	<0.001
Mean systolic blood pressure (mmHg)	146.1±35.8	147.4±34.8	144.8±36.7	0.016
Mean diastolic blood pressure (mmHg)	83.1±22.4	85.4±21.7	80.9±23.0	<0.001
Median B-type natriuretic peptide (pg/mL)	677 (350–1,220)	562 (298–981)	848 (439–1,490)	<0.001
Mean blood urea nitrogen (mg/dL)	25.2±18.6	18.7±15.5	31.8±19.2	<0.001
Mean serum creatinine (mg/dL)	1.15±0.52	0.80±0.18	1.50±0.51	<0.001
Mean eGFR (mL/min/1.73 m^2^)	51.9±21.6	68.9±15.9	34.8±9.7	<0.001
Mean serum sodium (mEq/L)	139.4±4.3	139.6±4.2	139.3±4.3	0.023
Mean hemoglobin (g/dL)	12.2±2.6	12.7±2.4	11.6±2.7	<0.001
Median total bilirubin (mg/dL)	0.8 (0.5–1.1)	0.8 (0.6–1.2)	0.7 (0.5–1.1)	<0.001

Data are expressed as mean ± standard deviation, as number (percentage), or as median (interquartile range).

eGFR, estimated glomerular filtration rate; HF, heart failure; ICD, implantable cardioverter-defibrillator; EF, ejection fraction; NYHA, New York Heart Association.

Before admission to the hospital and with the exception of digitalis, most types of medication, including diuretics, angiotensin-converting-enzyme inhibitors, angiotensin receptor blockers, calcium-channel blockers, beta blockers, nitrate, and statins, were more frequently used by patients with renal dysfunction than those without renal dysfunction. Although vasodilator use was similar in both groups during hospitalization, the use of intravenous diuretics and inotropes was significantly higher in patients with renal dysfunction. Non-pharmacologic management, including non-invasive or invasive positive-pressure ventilation, was similar for both groups, except for the application of revascularization therapy, which was more commonly used in patients without renal dysfunction (Table 2).

**Table 2:** Management of patients with and without renal dysfunction.

	Total	eGFR>50 mL/min/1.73 m^2^	eGFR≤50 mL/min/1.73 m^2^	
	(N = 4,321)	(n = 2,171)	(n = 2,150)	p-value
Intravenous therapy				
Diuretics, n (%)	3,306 (76.5)	1,622 (74.7)	1,684 (78.3)	0.005
Vasodilators, n (%)	3,392 (78.5)	1,708 (78.7)	1,684 (78.3)	0.781
Inotropes, n (%)	676 (15.6)	290 (13.4)	386 (18.0)	<0.001
In-hospital management				
Oxygen supplementation, n (%)	2,736 (63.3)	1,361 (62.7)	1,375 (64.0)	0.355
NIPPV, n (%)	1,012 (23.4)	501 (23.1)	511 (23.8)	0.592
Intubation, n (%)	259 (6.0)	117 (5.4)	142 (6.6)	0.094
Revascularization, n (%) therapy	348 (8.1)	202 (9.3)	146 (6.8)	0.002
Valve replacement, n (%)	98 (2.3)	66 (3.0)	32 (1.5)	<0.001
Outpatient medications before admission				
Loop or thiazide diuretics, n (%)	2,037 (47.1)	779 (35.9)	1,258 (58.5)	<0.001
ACE-I or ARB, n (%)	2,043 (47.3)	854 (39.3)	1,189 (55.3)	<0.001
Calcium-channel blockers, n (%)	1,192 (27.6)	528 (24.3)	664 (30.9)	<0.001
Beta blockers, n (%)	1,428 (33.0)	571 (26.3)	857 (39.9)	<0.001
Digitalis, n (%)	556 (12.9)	288 (13.3)	268 (12.5)	0.432
Nitrate, n (%)	726 (16.8)	286 (13.2)	440 (20.5)	<0.001
Amiodarone, n (%)	188 (4.4)	53 (2.4)	135 (6.3)	<0.001
Statins, n (%)	993 (23.0)	430 (19.8)	563 (26.2)	<0.001
Length of hospital stay (days)				
Median (interquartile range)	20 (13–30)	19 (13–28)	21 (13–33)	<0.001
Mean ± SD	27±34	25±28	29±39	<0.001

Data are expressed as mean ± standard deviation (SD), as number (percentage), or as median (interquartile range).

eGFR, estimated glomerular filtration rate; NIPPV, non-invasive positive-pressure ventilation; ACE-I, angiotensin-converting-enzyme inhibitor; ARB, angiotensin II receptor blocker.

**Figure 3:**
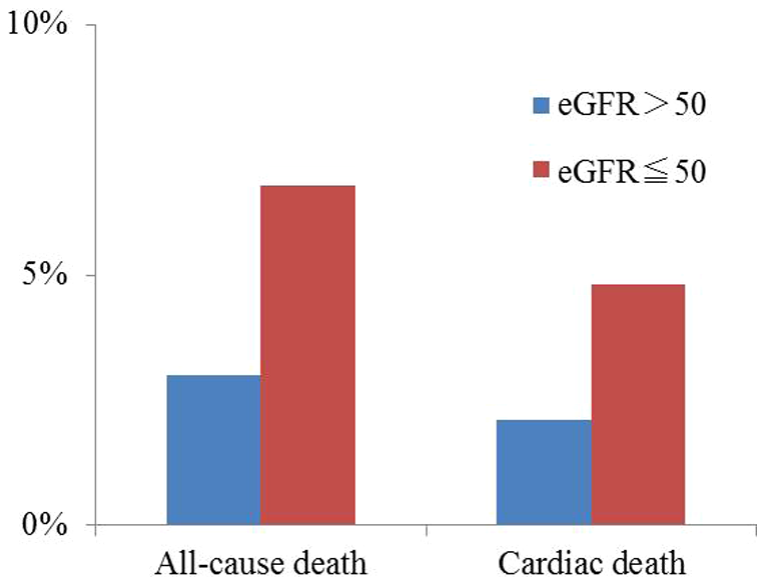
Relationship between the baseline estimated glomerular filtration rates and in-hospital mortality. eGFR, estimated glomerular filtration rate.

The all-cause death rate was significantly higher in patients with renal dysfunction at 6.8% compared with 3.0% for those without renal dysfunction. Furthermore, cardiac death rates were significantly higher in patients with renal dysfunction compared with those without renal dysfunction (4.8% vs. 2.1%, respectively, p<0.001) (Figure 3). Logistic regression analysis demonstrated that the presence of renal dysfunction was an independent predictor of all-cause death after adjustment for associated factors (OR: 2.36, 95% CI: 1.75–3.18, p<0.001).

**Figure 4:**
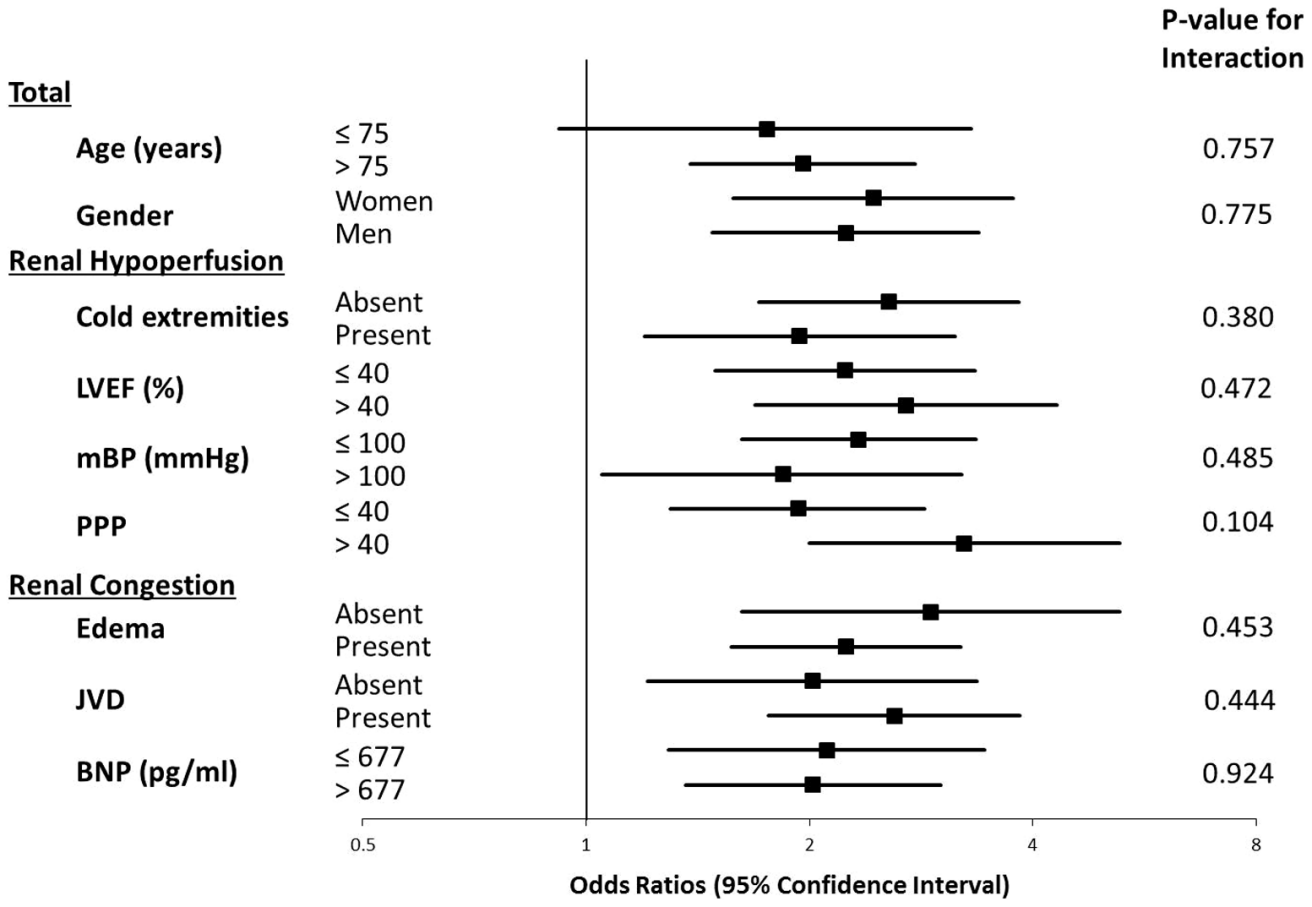
The prognostic impact of renal dysfunction in the prediction of all-cause mortality in relation to the underlying etiologic mechanisms. LVEF, left ventricular ejection fraction; mBP, mean blood pressure; PPP, proportional pulse pressure; JVD, jugular venous distension; BNP, brain natriuretic peptide.

To evaluate the prognostic impact of renal dysfunction in the context of the two underlying hemodynamic mechanisms, we performed logistic regression analyses on subgroups of patients with or without hypoperfusion-dominant characteristics (e.g., patients with cold extremities, low LVEFs, low mBPs, or low PPPs) and on subgroups of patients with or without congestion-dominant characteristics (e.g., edema, JVD or high BNP levels). As shown in Table 3, all-cause mortality was consistently higher in patients with renal dysfunction. The prognostic impact of renal dysfunction quantified using ORs, was similar across all of the subgroups, regardless of whether the clinical signs of hypoperfusion or congestion were present (Figure 4). The p-value for the interaction ranged from 0.104–0.924 and was always >0.05.

**Table 3:** All-cause mortality in different patient subgroups.

		Normal Renal Function			Renal Dysfunction		
		eGFR>50 mL/min/1.73 m^2^			eGFR≦50 mL/min/1.73 m^2^		
		No. of patients	No. of Events	No. of Events (%)	No. of Patients	No. of Events	No. of Events (%)
Total		2171	65	3.0%	2150	146	6.8%
Age (years)	≤75	1259	18	1.4%	850	21	2.5%
	>75	912	47	5.2%	1300	125	9.6%
Gender	Women	871	30	3.4%	949	76	8.0%
	Men	1300	35	2.7%	1201	70	5.8%
mBP (mmHg)	≤100	940	43	4.6%	1115	112	10.0%
	>100	1218	21	1.7%	1024	32	3.1%
PPP	≤42	1114	42	3.8%	955	67	7.0%
	>42	11044	22	2.1%	1184	77	6.5%
JVD	Absent	917	24	2.6%	815	42	5.2%
	Present	1088	37	3.4%	1158	97	8.4%
Edema	Absent	724	16	2.2%	663	41	6.2%
	Present	1423	46	3.2%	1464	102	7.0%
Cold extremities	Absent	1663	35	2.1%	1551	81	5.2%
	Present	409	26	6.4%	508	59	11.6%
BNP (pg/ml)	≤677	1182	28	2.4%	822	40	4.9%
	>677	830	35	4.2%	1175	97	8.3%
LVEF (%)	≤40	1181	39	3.3%	1120	77	6.9%
	>40	960	25	2.6%	993	67	6.7%

Abbreviation; eGFR, estimated glomerular filtration rate; mBP, mean blood pressure; PPP, proportional pulse pressure; JVP, juglur venous distension; BNP, brain natriuretic peptide; LVEF, left ventricular ejection fraction.

## Discussion

The major finding from this study was that renal dysfunction was significantly associated with an increased risk of in-hospital mortality in patients admitted with ADHF. Furthermore, this adverse effect of renal dysfunction on short-term outcomes remained the same, regardless of the underlying hemodynamic mechanism. The present study confirms previous findings from studies performed in Western countries that reported an association between baseline renal dysfunction and an increased risk of short-term mortality in patients admitted with ADHF [3], [4]

While various mechanisms have been proposed for renal dysfunction in patients admitted with ADHF, these mechanisms fall into two broad categories from the perspective of hemodynamics, namely renal hypoperfusion and renal congestion. A scientific statement to assess and grade congestion in acute heart failure has been proposed by the Acute Heart Failure Committee of the Heart Failure Association of the European Society of Cardiology [17]. Thus, if peripheral edema, JVD, and elevated BNP levels are the variables associated with congestion, then cold extremities and low LVEFs, mBPs, and PPPs could be the variables associated with hypoperfusion, because this type of renal dysfunction is attributed to reduced systemic perfusion. Using these definitions for each clinical profile, we demonstrated that, contrary to common belief, the typical physical findings indicative of renal hypoperfusion and renal congestion, including cold extremities and JVD, were more frequently observed in patients with renal dysfunction on hospitalization.

Traditionally, a reduction in renal blood flow, namely renal hypoperfusion, has been considered the main cause of renal dysfunction associated with ADHF. Although the precise mechanism that connects cardiac output with renal blood flow remains unclear in the context of ADHF, it is hypothesized that neurohormonal activation, for example via the renin-angiotensin system, results in afferent vasoconstriction, thereby reducing renal blood flow and hence the effective volume of circulating fluid, as is expected in patients with ADHF [7]. In contrast, recent studies have highlighted the association between an increased CVP and renal dysfunction or renal congestion. According to this hypothesis, elevated CVP is directly transmitted to the renal vein and increases renal perfusion pressure, which raises the interstitial intrarenal pressure and causes tubule collapse, leading to a decrease in GFR [18]. The association between a higher CVP and decreasing GFR has been demonstrated in several studies [10], [19]–[21]. Our study suggests that the resulting renal dysfunction could impact on patient outcomes, regardless of the etiology underlying the renal dysfunction.

In our study, patients' clinical presentation parameters and vital signs were primarily used to differentiate the underlying etiologic mechanisms of renal dysfunction; however, novel biomarkers could differentiate these mechanisms in more objective and reproducible fashion. Several novel biomarkers are emerging, and we evaluated their potential in the clinical settings. Among these biomarkers, soluble suppression of tumorigenicity 2 (sST2) could be a leading candidate. sST2, a member of the interleukin (IL)-1 receptor family, has been established as a predictor of mortality in the long-term follow-up of ADHF patients [22], [23]. As sST2 is a biomarker for cardiac remodeling and fibrosis, it may be more prominent in patients with hypoperfusion than in those with congestion.

Hypoperfusion has traditionally been considered the predominant cause of renal dysfunction in patients with ADHF [8]. However, a recent study reported that venous congestion may also be an important hemodynamic factor in this condition [10], and its impact has received strong attention. In turn, our study found the adverse impact of renal dysfunction on in-hospital outcomes to be consistent regardless of etiology. This finding has established the prognostic importance of renal dysfunction complicated with ADHF under any circumstances. Furthermore, our study also reconfirmed the adverse impact of renal dysfunction on in-hospital outcomes in the Asian population who have completely different clinical characteristics compared with the Western population. Previously, we demonstrated the key differentiating characteristics of heart failure patients in Western countries as compared with those in Asian countries [13]. Notably, we found an increased prevalence of patients with de novo heart failure and non-ischemic etiology in Japan versus in Western countries. Additionally, the length of hospital stay for this category of patients was much longer in Japan than in Western countries, probably owing to the differences in health insurance systems. All these complicating factors could potentially have mitigated the effect of eGFR.

### Study Limitations

Our study has several limitations. Firstly, the calculation of the GFR was originally developed for use in patients with chronic kidney disease whose renal functions are relatively stable; the applicability of this calculation for patients with ADHF has not been sufficiently validated. However, previous studies have demonstrated an association between reduced GFRs and adverse outcomes in patients with ADHF [24]–[26]. Our intent was to estimate the level of renal dysfunction in our study population, rather than to determine the precise renal function levels of these patients. Secondly, it could be argued that an invasive approach, such as right heart catheterization, should have been used to evaluate patients' hemodynamic profiles more precisely. However, we believe that evaluations based on accessible and non-invasive clinical measures, including vital signs, physical findings, laboratory markers, and echocardiograms, are relevant to clinical decision making. Furthermore, these non-invasive parameters reflect values assessed with an invasive modality, and they are considered sufficient substitutes for a more invasive approach [15]–[17]. Moreover, analyses based on these clinical measures may be more practical for patient assessments and more applicable in routine practice. Third, we could not evaluate the associations between renal dysfunction and long-term outcomes, because long-term follow-up data were not available for this study. Further study is needed regarding long-term assessments. Finally, hospital stays were much longer in the ATTEND registry than those reported from Western countries, which is associated with Japan's health insurance system [13], and in-hospital mortality in the data within the ATTEND registry might differ from its counterparts in other countries. However, a previous analysis of data from the ATTEND registry has shown that most sudden cardiac deaths occurred within 14 days of admission [27], therefore a hospital stay of less than 7 days might be too short to accurately evaluate short-term outcomes. From this perspective, our results may reflect short-term mortality more precisely.

### Conclusions

In conclusion, baseline renal dysfunction was significantly associated with in-hospital mortality in patients admitted with ADHF. The prognostic impact of renal dysfunction was the same, regardless of its underlying etiologic mechanism.

## Supporting Information

Appendix S1 **ATTEND Study Investigators.**
(DOC)
