# Supplementary material for: Prognostic Impact of Renal Dysfunction Does Not Differ According to the Clinical Profiles of Patients: Insight from the Acute Decompensated Heart Failure Syndromes (ATTEND) Registry
Source: PLoS One. 2014 Sep 8;9(9):e105596. doi: 10.1371/journal.pone.0105596 (PMC4157767; doi:10.1371/journal.pone.0105596)
Supplement: Appendix S1 — ATTEND Study Investigators. (DOC) [file pone.0105596.s001.doc]

**APPENDIX**

**ATTEND Scientific Advisory Committee:** Teruo Takano (Chair, takanot@nms.ac.jp), Hiroshi Kasanuki, Keiji Tanaka, Kyoichi Mizuno.

**Data and Safety Monitoring Board:** Tomomi Meguro, Toshihiko Sato.

**Endpoint Committee:** Masatoshi Kawana, Jun Nejima.

**Steering Committee:** Naoki Sato, Katsuya Kajimoto, Kuniya Asai, Masayuki Mizuno, Dai Yumino, Yuichiro Minami, Koji Murai, Ryo Munakata, Yasushi Sakata, Takehiko Keida, Toshiyuki Aokage.

**Statistical Analysis and Data Center:** Katsunori Shimada (STATZ Institute Inc., Tokyo, Japan).

**ATTEND Study Investigators**

**Nippon Medical School:** Naoki Sato, Kuniya Asai, Ryo Munakata, Toshiyuki Aokage, Asuka Yoshida; **Tokyo Women’s Medical University:** Yuichiro Minami, Dai Yumino, Masayuki Mizuno, Erisa Kawada, Kentaro Yoshida, Yuri Ozaki, Tomohito Kogure, Shintaro Haruki; **Saitamaken Saiseikai Kurihashi Hospital:** Masayuki Mizuno; **Aoyama Hospital, Tokyo Women’s Medical University:** Katsuya Kajimoto; **Saiseikai Kumamoto Hospital:** Koichi Nakao, Tadashi Sawamura, Toshiaki Nuki; **Toyota Memorial Hospital:** Ryoji Ishiki, Shigeki Yokota; **Tokushima Prefectural Central Hospital:** Hiroyuki Fujinaga, Takashi Yamamoto, Kenji Harada, Akihiro Saito, Norihito Kageyama, Takanobu Okumura; **Chiba Hokusoh Hospital, Nippon Medical School:** Noritake Hata, Koji Murai, Ayaka Nozaki; **Shizuoka Medical Center:** Hidekazu Kawanaka, Jun Tanabe; **Hyogo Prefectural Amagasaki Hospital:** Yukihito Sato; **Oji General Hospital:** Katsuhisa Ishii, Hitoshi Oiwa, Tomoaki Matsumoto, Daisuke Yoshida, Nobuo Kato; **Showa University Fujigaoka Hospital:** Hiroshi Suzuki, Nobuyuki Shimizu; **Edogawa Hospital:** Takehiko Keida, Masaki Fujita, Kentaro Nakamura, Toshiya Chinen, Kentaro Meguro, Tatsuro Kikuchi, Toshiyuki Nishikido, Marohito Nakata, Tatsuya Yamashita, Masaya Nakata; **Kurashiki Central Hospital:** Akitoshi Hirono, Kazuaki Mitsudo, Kazushige Kadota, Noriko Makita, Nagisa Watanabe; **Hyogo College of Medicine:** Masaaki Kawabata, Kenichi Fujii; **Yamaguchi University:** Shinichi Okuda, Shigeki Kobayashi; **Fukui Cardiovascular Center:** Ikuo Moriuchi, Kiyo-o Mizuno, Kazuo Osato, Tatsuaki Murakami, Yoshifumi Shimada, Katsushi Misawa, Hiromasa Kokado, Takashi Fujita, Yoshitomo Fukuoka, Syu Takabatake; **Tokyo Medical University Hospital:** Yoshifumi Takata, Manabu Miyagi, Nobuhiro Tanaka, Akira Yamashina; **Yatsu Hoken Hospital:** Shinji Sudo; **Shonan Dai-ichi Hospital:** Koichi Shimamura, Michitaka Nagashima; **Komatsu Municipal Hospital:** Tomoya Kaneda, Kosei Ueda, Hiromasa Kato, Toshinori Higashikata; **Sendai Cardiovascular Center:** Kanichi Fujimori, Hiroshi Kobayashi, Shinya Fujii, Masahiro Yagi, Yuri Ozaki, Jyunko Takaki; **Gunma Prefectural Cardiovascular Center:** Eiji Yamashita, Takuji Toyama; **Seirei Hamamatsu General Hospital:** Tetsuo Hirata, Toshiaki Oka; **Osaka City General Hospital:** Ryushi Komatsu, Akira Itoh, Takahiko Naruko, Yukio Abe, Eiichirou Nakagawa, Atsuko Furukawa, Naoto Kinou, Shoko Uematsu, Isao Tabuchi; **Suwa Central Hospital:** Taku Imai; **Kyushu University Hospital:** Takafumi Sakamoto, Koji Todaka; **Nagasaki University Hospital of Medicine and Dentistry:** Yuji Koide, Koji Maemura; **Tokai University School of Medicine:** Koichiro Yoshioka; **Fukushima Medical University:** Akiomi Yoshihisa, Takamasa Sato, Yasuchika Takeishi; **Yokohama City University Medical Center:** Toshiaki Ebina, Kazuo Kimura, Masaaki Konishi; **Tottori University Hospital:** Masahiko Kato, Yoshiharu Kinugasa, Katsunori Ishida, Shinobu Sugihara, Kiyotaka Yanagihara; **Asahikawa Medical College:** Toshiharu Takeuchi, Motoi Okada, Naoyuki Hasebe; **Showa University School of Medicine:** Tetsuo Sakai, Taku Asano, Yoshino Minoura, Tsutomu Toshida, Takatoshi Sato, Yuya Yokota, Seita Kondo; **Osaka University Hospital:** Yasushi Sakata, Issei Komuro, Kinya Otsu, Shizuya Yamashita, Yoshihiro Asano; **Kitamurayama Hospital:** Asuka Yoshida; **Sensoji Hospital:** Katsuya Kajimoto; **Osaka Police Hospital:** Kazunori Kashiwase, Yasunori Ueda; **Komaki City Hospital:** Taizo Kondo, Katsuhiro Kawaguchi, Akinori Sawamura; **Fukuoka Wajiro Hospital:** Taro Saito, Toru Higa, Hiroo Noguchi, Yoko Yanagita, Keita Nakamura, Tomo Komaki; **Saitama Medical University International Medical Center:** Toshihiro Muramatsu, Tomomi Koizumi, Yoshie Nakajima, Toshihiko Kikutani, Yoshifimi Ikeda, Toru Tamaki, Shuhei Funada, Harumi Ogawa; **Saiseikai Nagasaki Hospital:** Koichiro Sakuragawa; **Keio University School of Medicine:** Shun Kohsaka; **Saiseikai Futsukaichi Hospital:** Shin-ichi Ando, Toshiaki Kadokami, Eiko Ishida, Katsumi Ide; **Sakurabashi Watanabe Hospital:** Yoshiharu Higuchi, Motoko Uehara, Yohei Sotomi; **Nagoya City University Graduate School of Medical Sciences:** Toshihiko Goto, Nobuyuki Ohte; **Tohoku University Hospital:** Masanobu Miura, Nobuyuki Shiba, Kotaro Nochioka, Hiroaki Shimokawa; **Nippon Steel Yawata Memorial Hospital:** Shiro Ishihara, Tokushi Koga, Shinichiro Fujishima, Shigeru Kaseda, Yoshie Haga; **St. Marianna University School of Medicine Hospital:** Keisuke Kida; **Tokyo Women's Medical University Yachiyo Medical Center:** Kazuho Kamisihima; **Okinawa Prefectural Nannbu Medical Center & Children's Medical Center:** Makiko Nakamura, Osahiko Sunagawa, Takafumi Miyara, Youji Taba, Takashi Touma, Osamu Shinjo; **Jichi Medical University:** Yoshioki Nishimura, Kazuomi Kario, Hayato Shimizu; **Nippon Medical School Musashi Kosugi Hospital:** Takahiro Uchida, Ken-ichi Amitani, Naoki Sato.
